# Clinical features and genetic characteristics of two Chinese pedigrees with fatal family insomnia

**DOI:** 10.1080/19336896.2019.1617027

**Published:** 2019-05-23

**Authors:** Runcheng He, Yacen Hu, Lingyan Yao, Yun Tian, Yafang Zhou, Fang Yi, Lin Zhou, Hongwei Xu, Qiying Sun

**Affiliations:** a Department of Neurology, Xiangya Hospital, Central South University, Changsha, Hunan, China; b National Clinical Research Center for Geriatric Disorders, Changsha, Hunan, China; c Department of Geriatrics, Xiangya Hospital, Central South University, Changsha, China

**Keywords:** Fatal family insomnia, clinical features, genetic characteristics, pedigree, PRNP, gene mutation, thalamus

## Abstract

**Background**: Fatal familial insomnia (FFI) is a rare autosomal-dominant inherited prion disease characterized clinically by severe sleep disorder, motor signs, dysautonomia and abnormal behaviour. FFI is caused by a missense mutation at codon 178 of the prion protein gene (PRNP). Our study is aimed to explore typical clinical and genetic features of two Chinese pedigrees with FFI and review the related literatures.

**Methods**: Two FFI cases with family histories were recruited in our study. The main clinical features, genetic features and possible pathophysiologic mechanisms of these two FFI cases were analysed.

**Results**: The foremost symptoms seemed to be sleep disturbances and psychosis. Progressive sympathetic symptoms, movement disturbances and memory loss were frequently observed as well. Electroencephalography (EEG) showed a minor slowing without periodic triphasic waves. Polysomnography (PSG) showed reduction in total sleep time and disturbance of sleep-related respiratory. Brain magnetic resonance imaging (MRI) did not reveal obvious abnormality. Genetic analysis disclosed the prion protein gene mutation at codon 178 (D178N), with methionine (Met) homozygosity at the polymorphic position 129 (Met129Met).

**Conclusions**: The major clinical features of Chinese FFI are sleep dysfunction, psychiatric symptoms and sympathetic symptoms. Our patients have similar clinical characteristics as that of the typical FFI cases.

## 1. Introduction

Fatal familial insomnia (FFI) first reported in 1986 [1], as well as Gerstmann-Sträussler-Scheinker disease (GSS) and familial Creutzfeldt-Jakob disease (fCJD), are different subtypes of human genetic prion-protein (PrP) diseases [2]. FFI is a rare neurodegenerative autosomal-dominant inherited PrP disease linked to a prion protein gene (PRNP) missense mutation at codon 178 (D178N) coupled with the presence of a methionine (Met) at the polymorphic codon 129 on chromosome 20 [3].

FFI has been reported in more than 100 pedigrees worldwide, with an average age at onset of disease of around 50 years and a duration of the disease varies from 8 to 72 months [4]. The main clinical manifestations of it include severe sleep disorder, motor signs, dysautonomia and abnormal behaviour. Pathologically, the typical neurodegeneration in FFI is predominantly in the thalamus especially in the anterio-ventral and medio-dorsal nuclei where there is severe neuron loss and astrocytic gliosis [5]. But routine brain computed tomography (CT) and magnetic resonance imaging (MRI) usually reveal nonspecific features.

In this study, we collected information about nine Chinese patients with FFI from two pedigrees and the detailed clinical features, familial characteristics and genetic findings of these nine patients were comparably analysed.

## 2. Results

### 2.1. Clinic features of Chinese patients with FFI

A total of 47 family members of the first proband and 23 family members of the second proband were retrospectively or directly investigated. In the first pedigree, the cause of death of the proband’s grandparents and uncles is unclear. Two family members in the proband’s generation and two in the next generation died from similar neurological disorders. In the second pedigree, the cause of death of the proband’s grandparents is also uncertain. His brother and two sisters died from similar neurological disorders. A total of nine family members (five males and four females) were judged to be suspect FFI cases. The median age of onset of FFI was 49 years old (range from 17 to 60 years old) and total clinical duration ranges from 6 to 20 months. Among the most prominent symptoms, sleep disturbances, including insomnia, sleep-related dyspnoea, sleep-related involuntary movements and laryngeal stridor appeared in all nine of the cases (9/9). In addition, rapidly progressive dementia (RPD) and psychiatric symptoms (9/9) were the most prominent neuropsychiatric symptoms, and they were observed in almost all of the cases as well. Ataxia (7/9), pyramidal sign (5/9) and parkinsonism (3/9) were also observed. From early to the late of clinical course, progressive sympathetic symptoms were noticed in eight of the cases (8/9), the most common of which was excessive sweating, followed by hypertension (7/9). Irregular breathing (2/9) and tachycardia (3/9) are relatively rare. Cerebral spinal fluid (CSF) biochemical tests of all patients were basically normal. Electroencephalogram (EEG) examination did not record periodic sharp waves in any case, except for generalized slow waves in several patients (5/5). MRI only showed nonspecific abnormality including mild cerebral cortical atrophy and mild hyperintense signals in the subcortical and periventricular area. Polysomnography (PSG) analysis showed a decrease in the sleep spindles and K complexes in four cases (4/5), accompanied by a drastic reduction in total sleep time. Moreover, they were all identified with sleep apnoea syndrome. Five patients underwent a 18-fluoro-deoxy-glucose-positron emission tomography (PET)/CT scan, and three of them showed reduced glucose uptake in the bilateral thalamus, basal ganglia and subcortical areas. The main clinical characteristics of these nine FFI patients are summarized in Tables 1 and 2. The general clinical manifestations of these FFI cases and the comparison with other cases were summarized in Table 3 [6–8].

**Table 1.** The comparison of the main clinical features of five FFI patients in the first pedigree. RPD: rapidly progressive dementia. +: symptom/sign observed, −: symptom/sign not observed.

Parameters	Case 1	Case 2	Case 3	Case 4	Case 5
Gender	Male	Male	Female	Male	Male
Age at onset (years)	60	49	45	22	17
Duration of disease (months)	13	6	8	10	11
Foremost symptoms	Sleep loss	Insomnia	Psychiatric symptoms	Insomnia	Psychiatric symptoms
Cluster A-sleep-related symptoms					
Insomnia	+	+	+	+	+
Sleep-related involuntary movement	+	+	+	+	+
Sleep-related dyspnoea	+	+	+	+	+
Laryngeal stridor	+	+	+	+	+
Cluster B-neuropsychiatric symptoms					
RPD	+	+	+	+	+
Psychiatric symptoms	+	+	+	+	+
Ataxia	+	+	+	+	−
Pyramidal sign	+	−	+	−	+
Parkinsonism	−	+	−	−	−
Cluster C-progressive sympathetic symptoms					
Hypertension	+	+	+	−	−
Sweating	+	+	+	+	−
Tachycardia	+	+	−	−	−
Irregular breathing	−	−	+	−	−
EEG changes (periodic sharp waves)	N.A.	−	N.A.	−	−
MRI changes	N.A.	−	N.A.	−	−
PET/CT changes (hypometabolism)	N.A.	+	N.A.	+	−
Polysomnography changes (REM, efficiency and deep sleep reduction)	NA	+	NA	+	−

**Table 2.** The comparison of the main clinical features of four FFI patients in the first pedigree.

Parameters	Case 6	Case 7	Case 8	Case 9
Gender	Female	Male	Female	Female
Age at onset (years)	49	44	55	57
Duration of disease (months)	20	7	18	10
Foremost symptoms	Insomnia	Insomnia	Sleep loss	Sleep loss
Cluster A-sleep-related symptoms				
Insomnia	+	+	+	+
Sleep-related involuntary movement	+	+	+	+
Sleep-related dyspnoea	+	+	+	+
Laryngeal stridor	+	+	+	+
Cluster B-neuropsychiatric symptoms				
RPD	+	+	+	+
Psychiatric symptoms	+	+	+	+
Ataxia	+	−	+	+
Pyramidal sign	+	−	−	+
Parkinsonism	−	+	−	+
Cluster C-progressive sympathetic symptoms				
Hypertension	+	+	+	+
Sweating	+	+	+	+
Tachycardia	−	−	+	−
Irregular breathing	−	−	−	+
EEG changes (slowing waves)	NA	−	−	NA
MRI changes	NA	−	−	NA
PET/CT (hypometabolism)	NA	+	−	NA
Polysomnography changes (REM, efficiency and deep sleep reduction)	NA	+	+	NA

RPD: rapidly progressive dementia; +: symptom/sign observed; −: symptom/sign not observed.

**Table 3.** The comparison of clinical manifestations between our FFI patients and previous cases.

		Our cases	Manetto, V [1]	Gao C [2]	Wu L [3]
Gender(male: female)		5:4	4:3	5:5	2:3
Median age at onset (years)		49	48	38	46.4
Duration of disease (months)		10	9	9.5.	11
Sleep-related symptoms		9/9	7/7	10/10	5/5
RPD		9/9	NA	8/10	5/5
Psychiatric symptoms		9/9	NA	NA	4/5
Ataxia		8/9	6/7	5/10	4/5
Pyramidal sign		5/9	NA	8/10	2/5
Parkinsonism		3/9	NA	3/10	1/5
Progressive sympathetic symptoms		8/9	5/7	10/10	5/5
14-3-3 protein in CSF (positive)		0/2	NA	5/8	0/5
EEG changes (periodic sharp waves)		0/5	1/7	0/10	0/5
PET/CT changes (hypometabolism)	3/5		NA	NA	2/2
Polysomnography changes (REM, efficiency and deep sleep reduction)	4/5		3/7	NA	5/5

### 2.2. PRNP analyses

**Figure 3.**
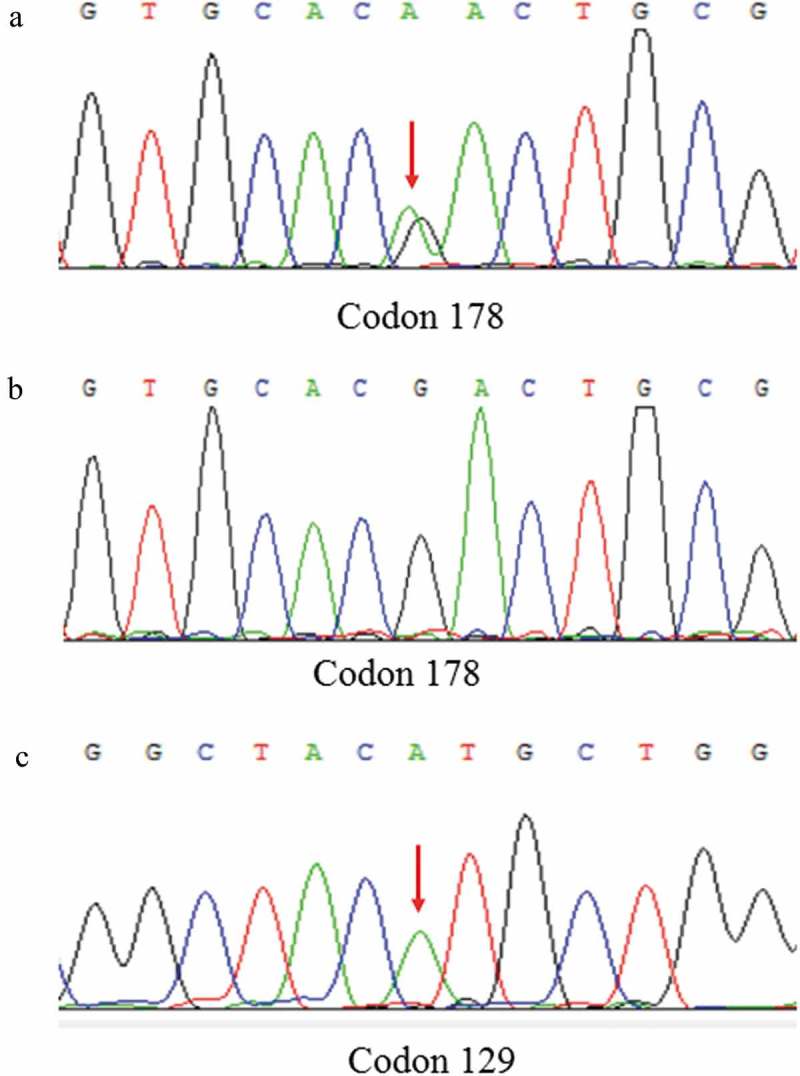
DNA sequence at codon 178 of PRNP gene from the patient (A) and a normal case (B). The red arrow indicates the c.532G > A mutation causing a substitution of GAC (asparagine) by AAC (aspartic) at codon 178 of the protein. DNA sequence at codon 129 of PRNP gene from the patient (C) shows Met at codon 129 of the PRNP gene.

PRNP sequences revealed that nine definite FFI patients had mutation (G to A) at the position of nt 532 in a PRNP allele, which is predicted to change aspartic acid (Asp) to asparagine (Asn) at codon 178 of the protein, accompany with Met/Met homozygosity at the polymorphic 129 codon, consistent with the genetic characteristics of FFI. In addition, the first proband’s son and brother also contain D178N mutation with M129M, but they remain healthy. The results of sanger sequencing were shown in Figure 3.

## 3. Discussion

It is well known that prion diseases, a fatal neurodegenerative disorder, differ from the viral or bacterial infectious diseases, because prion protein (PrP) has no DNA or RNA. The latest incidence of human prion diseases, caused by the misfolding of PrP, is about 1.2 per 1 million [9]. Although most patients have no definable cause, about 10–15% of patients are caused by point mutations or insertions of octapeptide repeats in PRNP, and they are dominantly inherited. Up to date, at least 30 pathogenic mutations of the PRNP worldwide have led to familial prion diseases [10]. Based on clinical and pathological features, genetic prion diseases are classified into three phenotypes: FFI, genetic Jakob–Creutzfeldt disease (gCJD) and Gerstmann–Sträussler–Scheinker (GSS). Genetically, FFI is correlated with a point mutation at codon 178 of PRNP resulting in a substitution of Asn for Asp (D178N) [11]. This mutation will decrease the thermodynamic stability of PrP, which can be involved in neuronal protection, cell signalling and control of the circadian system, by breaking the salt bridge [12]. D178N mutation can lead to both FFI and CJD. Their clinical phenotype depends on the presence of Met or valine (Val) at codon 129. FFI is correlated to the Met allele [13]. FFI patients with heterozygous (Met-Val) mutation have a longer mean survival time than those with homozygous (Met-Met) [3]. However, the 129 amino acid polymorphisms of the PRNP gene vary widely among different races. The frequencies of the 129 Met allele in the Chinese populations are much higher than those of the European. Consequently, FFI is the most common genetic prion disease in the Chinese population [9,14,15].

Here, we report nine FFI cases in two Chinese families. There is no significant gender difference in the incidence of FFI. They both have typical clinical features similar to previous descriptions of FFI [16]. Sleep disturbances, such as insomnia and sleep loss, are the common foremost symptoms of the Chinese FFI patients. Early PSG also shows the remarkable sleep abnormalities, including the loss of sleep spindles and K complexes. Several studies have confirmed that sleep spindles are produced by the thalamus [6,17]. As a result, the reduction of the sleep spindles and K complexes indicates a decline in thalamic function [18]. Furthermore, 18-fluoro-deoxy-glucose-PET/CT detected prominent hypometabolism in bilateral thalami, which were consistently reported in previous publications [4,19]. Early pathological changes of FFI may be predominantly limited to the thalamus and adjacent areas. It has been suggested that the neurodegenerative process starts in the thalamus approximately 13 to 21 months prior to the onset of clinical manifestations of the disease [20]. In several postmortem studies of FFI, thalamic neuronal loss and astrocytic gliosis are the main early alterations, without concomitant spongiform change. The most vulnerable nuclei are the mediodorsal and anterior ventral nuclei of the thalamus, the pulvinar and the inferior olives [21]. Consequently, severe insomnia early in FFI is caused by the prominent damage to the thalamus and the pulvinar. Furthermore, psychiatric symptoms, such as delusions, are also related to the putamen and thalamus volume loss [22]. We also observed characteristic sleep-related symptoms in each FFI case, including sleep-related involuntary movements and laryngeal stridor. Abnormal involuntary movements have been confirmed to be associated with the thalamus [23]. Laryngeal stridor may be caused by passive laryngeal narrowing due to the contraction of vocal cord adductor muscles during inspiration [24]. Sympathetic hyperactivities, for example, excessive sweating and hypertension, are also very common clinical manifestations. These characteristic symptoms may be associated with the selective involvement of the thalamus as well. In the pathophysiology of severe sympathetic hyperactivity, rapid progression of these symptoms may be related to bilateral thalamic damage [25]. Severe persistent atrophy of these nuclei can distinguish FFI from other prion diseases. In addition, it has been suggested that cortico-basal ganglia loop may control sleep–wake cycle [26]. Similarly, we found that bilateral subcortical areas and basal ganglia were damaged in FFI patients by PET/CT scan.

The diagnosis of FFI is quite complicated at present. Genetic testing is very beneficial to the diagnosis of FFI. However, some patients with the D178N/Met129Met genotype do not have any clinical symptoms in the early stages of the disease. Typical manifestations may appear gradually over time, especially in 21–62 years [27]. Therefore, it is necessary to find a robust biomarker test for early diagnosis. It has been reported that combination of biochemical biomarkers, neuroimaging and genetic screening are necessary for the early and accurate diagnosis of neurodegenerative diseases, such as Alzheimer’s disease (AD) and Parkinson’s disease [28–30]. Although genetic testing was strongly recommended. We also need to make great efforts to explore potential biomarkers, including PSG, EEG and single-photon emission computed tomography (SPECT) scans to improve the diagnostic accuracy of FFI.

In our studies, five patients were treated with alprazolam and olanzapine. But they showed poor response to sedative drugs. Furthermore, it was reported that benzodiazepines and neuroleptics may lead to the clinical worsening [31]. However, phenothiazine, an antipsychotic drug, appears to be effective in short-term therapy. Because it can cure psychotic symptoms and improve sleep patterns. Melatonin supplements and mind-body therapies can also induce sleep, which may play a therapeutic role in the early stages of the disease [32,33].

## 4. Conclusions

FFI is a hereditary autosomal-dominant prion disease. The major clinical symptoms of Chinese FFI are sleep disturbances, accompanied by neuropsychiatric symptoms and progressive sympathetic symptoms. Insomnia, sleep loss and psychotic symptoms are the clinical manifestations of early-stage FFI. These manifestations may result from the neuronal loss in the inferior olives and anterior medial thalamus. Currently, we have not found a cure for diseases. We hope that we can find more biomarkers to establish an early FFI diagnosis, especially for target screening of the D178N/Met129Met mutations in PRNP.

## 5. Material and methods

### 5.1. Patients

**Figure 1.**
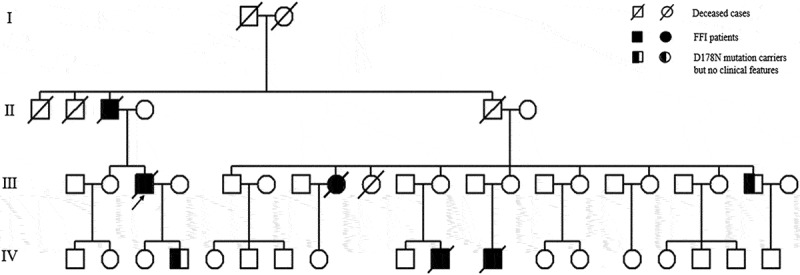
Family tree of the first pedigree. Patient III-3 is the proband. Patient Ⅱ-3, Patient III-8, Patient Ⅳ-10 and Patient Ⅳ-11 show similar clinical manifestations as the proband, carrying PRNP D178N mutation. Patient III-20, the proband’s brother; PatientⅣ-5, the proband’s son are clinically healthy carriers of the PRNP D178N mutation.

**Figure 4.**
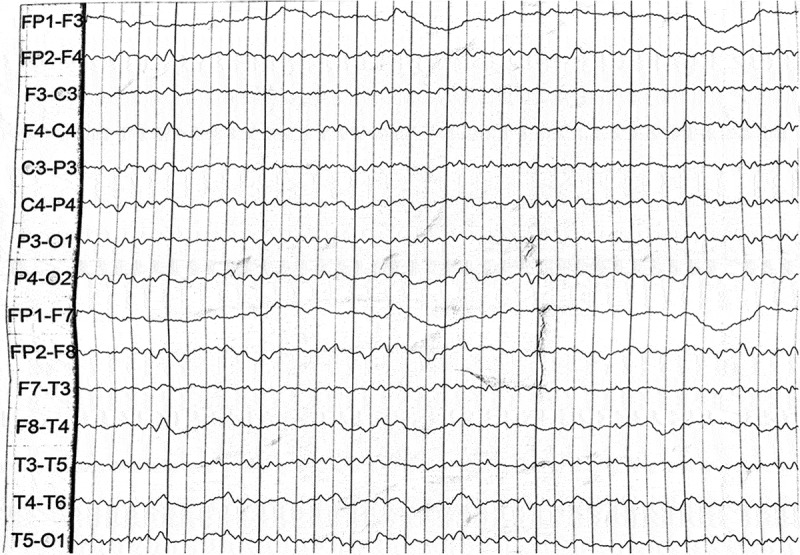
EEG showed slowing waves.

**Figure 5.**
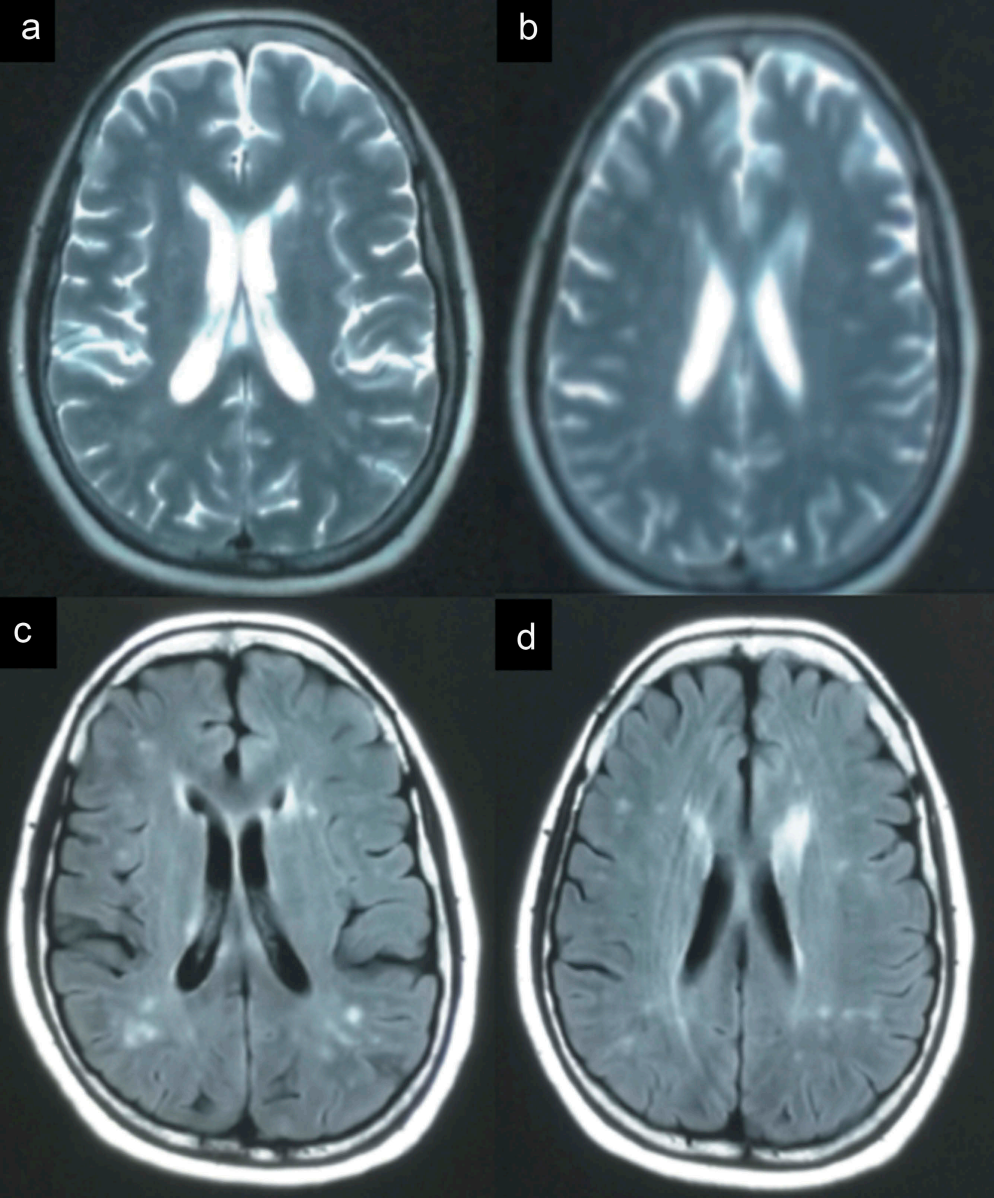
The imaging of the patient. MRI showed abnormal signals in the bilateral frontal subcortical areas and the periventricular area of the lateral ventricles.

Two patients were referred to the Department of Geriatrics, Xiangya Hospital of Central South University in 2018. The first proband was a 50-year-old male with a 6-month history of sleep dysfunction and psychotic behaviour disturbance. He complained initially of insomnia, nocturnal hallucination without a known cause. Meanwhile, he started to engage in involuntary movement such as kicks and grasps during her sleep. Two months later, he was found to have shrill laryngeal sounds and unconscious talking while inhaling during sleep. Two months before admission, a rapid deterioration of his sleep disturbance was observed. He could not fall asleep all night. During the daytime, intermittent peculiar personality changes, memory loss, unsteady gait and autonomic hyperactivity, including salivation, excessive sweating and hypertension, were noticed. Eventually, he was unable to do housework. His family history showed that his father, his cousins and nephews had very similar clinical manifestations as him. The pedigree is summarized in Figure 1. Upon admission, physical examination revealed psychotic symptoms and confusion. He exhibited significant impairments in temporal and spatial orientation, calculation abilities and recent memory. He had bulbar palsy, dysarthria, stridor, gait ataxia and involuntary movement with normal muscle tone. Pathologic reflexes were negative on both sides. Blood examinations including blood routine, liver function, chemistry, electrolytes, thyroid function, lipid profile, ceruloplasmin, tumour markers, paraneoplastic antibodies and autoimmune encephalitis antibodies were normal. CSF examinations, including glucose, protein, cell count and autoimmune indexes, were normal. 14-3-3 protein in CSF was negative as well. EEG showed generalized slowing without sharp-wave complexes (Figure 4). PSG showed a significant reduction in the sleep spindles and K complexes. Brain MRI indicated diffuse mild high signal intensity in the bilateral frontal subcortical areas and the periventricular area of the lateral ventricles (Figure 5). The blood samples of the patient and his family members were obtained with informed consent. Then, we amplified the extracted DNA for the coding region of PRNP and analysed the genotype at codon 129 and 178 of PRNP. Two weeks later, our patient died and his total duration of disease was about 7 months.

**Figure 2.**
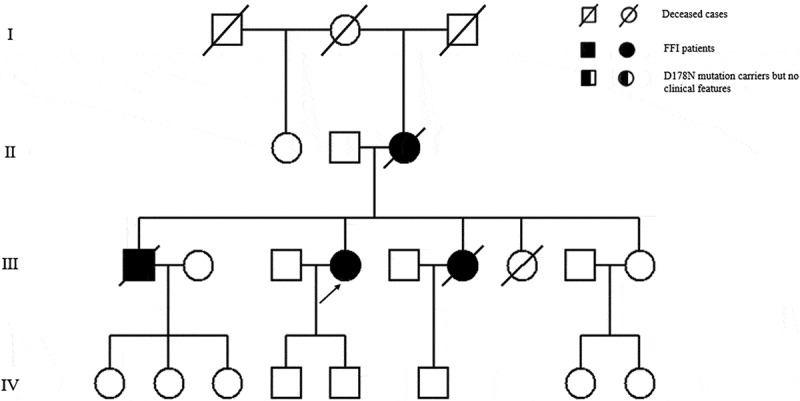
Family tree of the second pedigree. Patient III-4 is the proband. Patient Ⅱ-3, Patient III-1 and Patient III-6 show similar clinical features as the proband, carrying PRNP D178N mutation.

The second proband, a 56-year-old female, was admitted to our department with complaints of 15-month history of progressive sleep disturbance and 3-month history of abnormal behaviours. He had begun to suffer from sleep loss with total sleep time decreasing from 8 to 3 h per night 15 months ago. Nine months before admission, she had depressive, nervousness and anxiety symptoms. And she was unable to sleep even when feeling tired. Six months later, she developed worsening nocturnal psychotic mood disturbances and involuntary movement such as kicks, accompanied by amnesia, hallucinations, dysarthria and unbalanced gait. She took alprazolam, olanzapine and flupentixol, but it did not work. Eventually, she was unable to do housework as well. She was the fourth individual in her family to show the same clinical manifestations. The pedigree is summarized in Figure 2. And the other three members all died within two years after onset of disease. On admission, her cranial nerve examination was unremarkable. On the Mini-Mental Status Exam (MMSE), the patient scored 18 out of 30 and showed significant deficits in calculation and memory abilities. He had dysarthria and unsteady gait with normal muscle tone. Except for carcinoembryonic antigen (CEA), routine haematological and biochemical laboratory testing were unremarkable. CSF examinations, including 14-3-3 protein test, and long-term continuous video-EEG were within normal limits as well. PSG disclosed a severely reduced total sleep time and increased arousals. At the same time, it also identified sleep apnoea syndrome, characterized by obstructive in the non-rapid eye movement (NREM) stage. Brain MRI indicated diffuse mild high signal intensity in the bilateral frontoparietal subcortical areas, centrum semiovale and the periventricular area of the lateral ventricles. 18-Fluoro-deoxy-glucose-PET/CT showed hypometabolism in the bilateral subcortical areas, thalamus and basal ganglia.

The blood samples of him and his family members across three generations were also taken after obtaining informed consent.

### 5.2. Methods

Blood samples were obtained from two proband and from their relatives with informed consent. This study was approved by the Ethics Committee of Xiangya Hospital of Central South University. All subjects provided written consent to obtain blood samples for genetic examination. Experimental procedures consist of three steps. Step 1: library construction. Genomic DNA was extracted from peripheral blood and fragmented with standard procedure. Then, the fragments were ligated by adapters and purified. Step 2: polymerase chain reaction (PCR) amplification: forward primer: 5′-CAGAGCAGTCATTATGGCGAACCT-3′; reverse primer: 5′-AGACCTTCCTCATCCCACTATCAG-3′. All PCR reactions were performed as following conditions: initial denaturation at 94°C for 5 min; 30 cycles at 94°C for 40 s, 55°C for 40 s and 72°C for 40 s; and a final extension at 72°C for 10 min. The predicted size of PCR products was 759 bp. Step 3: sequencing: the resulting amplicons were screened for mutations in exons of PRNP via Sanger sequencing using an ABI 3700 instrument (Applied Biosystems).
